# YjbH Solubility Controls Spx in *Staphylococcus aureus*: Implication for MazEF Toxin-Antitoxin System Regulation

**DOI:** 10.3389/fmicb.2020.00113

**Published:** 2020-02-06

**Authors:** Olesya O. Panasenko, Fedor Bezrukov, Olga Komarynets, Adriana Renzoni

**Affiliations:** ^1^Service of Infectious Diseases, Department of Medical Specialties, University Hospital and Medical School of Geneva, Geneva, Switzerland; ^2^Department of Microbiology and Molecular Medicine, Faculty of Medicine, University of Geneva, Geneva, Switzerland; ^3^Department of Physics and Astronomy, The University of Manchester, Manchester, United Kingdom; ^4^Department of Cell Physiology and Metabolism, Faculty of Medicine, University of Geneva, Geneva, Switzerland

**Keywords:** YjbH, aggregation, *Staphylococcus aureus*, Spx, MazEF, toxin-antitoxin system, dormancy, antibiotic resistance

## Abstract

Bacterial cells respond to environmental stresses by modulating their gene expression and adjusting their proteome. In *Staphylococcus aureus*, selective degradation by ClpP protease eliminates damaged proteins and regulates the abundance of functional proteins such as many important stress-induced transcriptional regulators. Degradation by ClpP requires the unfolding activity of partner Clp ATPases, such as ClpX and ClpC, and assistance of substrate-specific adaptor proteins such as YjbH and TrfA. Herein, we demonstrated that YjbH is aggregated in response to growth stress stimuli, such as oxidative and antibiotic stresses. In consequence, its function as an adaptor protein is compromised. YjbH controls the degradation of the stress-induced transcriptional regulator, Spx. Aggregated YjbH cannot assist Spx degradation, which results in Spx accumulation. We discovered that depending on the stress stimulus, Spx can be soluble or insoluble, and, consequently, transcriptionally active or inactive. Therefore, Spx accumulation and solubility are key components governing activation of Spx-dependent genes. Spx positively regulates expression of a ClpCP adaptor protein TrfA. TrfA in turn is required for degradation of MazE antitoxin, the unstable component of the MazEF toxin-antitoxin system, that neutralizes the endoribonuclease activity of MazF toxin. Bacterial toxin-antitoxin systems are associated with dormancy and tolerance to antibiotics that are related to chronic and relapsing infections, and it is at present a key unresolved problem in medicine. MazF activity was linked to growth stasis, yet the precise environmental signals that trigger MazE degradation and MazF activation are poorly understood. Here we propose a model where YjbH serves as a sensor of environmental stresses for downstream regulation of MazEF activity. YjbH aggregation, soluble Spx, and TrfA, coordinately control MazE antitoxin levels and consequently MazF toxin activity. This model implies that certain stress conditions culminate in modulation of MazF activity resulting in growth stasis during *in vivo* infections.

## Introduction

Bacterial antibiotic resistance has been recognized as a worldwide problem with still few solutions. It may occur as a result of mutations in bacterial populations selected during antibiotic treatment. However, another important aspect of antimicrobial resistance is bacterial dormancy.

The first type of dormant bacteria tolerant to antibiotics was described in *Staphylococcus aureus* strains long ago (Bigger, 1944). Antibiotic treatment of a susceptible bacterial population kills the majority of the cells but induces the formation of non-dividing dormant bacteria that survive antibiotic challenge. Dormant bacteria are not genetically resistant and after removal of antibiotics, they can regrow and evolve into a susceptible population. The mechanism of entry into antibiotic tolerant state with later regrowth after antibiotic removal, may explain the clinical chronic and relapsing infections. This emphasizes the urgent need to understand the molecular pathways that lead to bacterial dormancy.

Toxin-antitoxin systems (TAS) are stress-inducible functional complexes where toxin component binds an antitoxin (Yamaguchi et al., 2011; Page and Peti, 2016). Toxin activity is inhibited by the antitoxin, that is typically an unstable protein susceptible to degradation by proteases. Antitoxin degradation leads to toxin activation that down-regulates central processes in the cell and may result in cell dormancy (Coussens and Daines, 2016). Different bacterial species enter into dormancy through activation of TAS that will interfere with replication (Maki et al., 1992; Aakre et al., 2013; Harms et al., 2015), inhibition of ribosomes (Castro-Roa et al., 2013; Van Melderen and Wood, 2017), cell wall synthesis (Mutschler et al., 2011), and cell division (Masuda et al., 2012; Mok et al., 2015).

**FIGURE 1:**
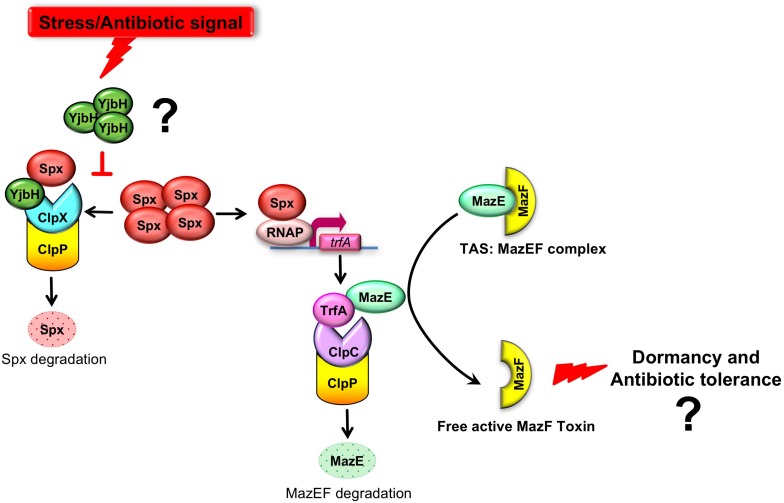
A model where YjbH serves as a sensor of environmental stress and downstream regulation MazEF activity. MazEF complex is composed of MazF toxin and MazE antitoxin which binds MazF and neutralizes MazF activity. MazE is cleaved by the ClpCP degradation module, where ClpC is a chaperone with unfolding activity and ClpP is a protease. MazE degradation is assisted by the adaptor protein TrfA, providing ClpCP specificity and facilitating MazE recognition. The *trfA* transcription is regulated by the redox sensitive transcriptional factor, Spx. In turn, Spx proteolysis is controlled by ClpXP proteolytic system and requires YjbH adaptor protein. We hypothesized (indicated by question mark) that in *S. aureus* YjbH aggregates and modulation of YjbH aggregation affects MazEF TAS through the YjbH-Spx-TrfA cascade in response to environmental stresses.

One of the best characterized TAS in *S. aureus* is MazEF, a type II TAS (Schuster and Bertram, 2016). It is found also in other clinically important bacteria (Mittenhuber, 1999; Nguyen et al., 2011; Schifano et al., 2013; Cho et al., 2017). Several studies were conducted to characterize the MazEF locus by studying its transcriptional activation and function (Donegan and Cheung, 2009; Fu et al., 2009; Zhu et al., 2009; Zorzini et al., 2011, 2014; Miyamoto et al., 2018). MazEF is composed of MazF toxin and its activity is modulated by the MazE antitoxin (Figure 1). Under normal growth conditions, high MazE level ensures formation of toxin-antitoxin complex and consequently, MazF inactivity (Fu et al., 2007). MazE is cleaved by the ClpCP degradation module, where ClpC is a chaperone with unfolding activity and ClpP is a protease. MazE degradation is assisted by the adaptor protein TrfA, providing ClpCP specificity and facilitating MazE recognition (Donegan et al., 2010, 2014). We previously showed that transcription of *trfA* is positively regulated by the transcriptional activator Spx (Jousselin et al., 2013). In *Bacillus subtilis*, Spx is a central regulator of the stress response. It binds to the alpha subunits of RNA polymerase, and regulates positively and negatively expression of many genes (Schafer et al., 2019). Spx protein levels are regulated by the proteolytic system ClpXP and assisted by the adaptor protein YjbH (Garg et al., 2009; Renzoni et al., 2011; Engman et al., 2012) (Figure 1). It has been reported that *Geobacillus thermodenitrificans* YjbH directly interacts with C-terminal end of *B. subtilis* Spx to accelerate Spx proteolysis by ClpX (Chan et al., 2012, 2014). Later the crystal structure of YjbH from *Geobacillus kaustrophilus*, a functional homologs of YjbH from *S. aureus*, bound to *B. subtilis* Spx was published (Awad et al., 2019). In *B. subtilis* it was demonstrated that YjbH is aggregated in response to environmental stresses, and it was proposed that via aggregation YjbH may control Spx levels (Engman and von Wachenfeldt, 2015). However, in *S. aureus* the regulation and properties of Spx and YjbH are poorly understood.

It has been reported that MazF toxin overexpression in *S. aureus* leads to growth stasis or growth arrest (Fu et al., 2009), raising the question whether MazF may be a potential regulator of bacterial dormancy and antibiotic tolerance. Several studies identified genome-wide targets of MazF trying to clarify its role in growth stasis (Fu et al., 2009; Zhu et al., 2009; Schuster et al., 2015; Culviner and Laub, 2018; Sierra et al., 2019). However, the link of MazEF to bacterial dormancy is still to be determined. The described metabolic effects of MazF have been observed under artificial overexpression of MazF. Presently, it is still unknown which environmental conditions, mechanism of sensing, and signal transmission lead to active and free MazF toxin.

We provide evidences that in *S. aureus* YjbH aggregates in response to different environmental stresses. Both YjbH aggregation and the stress conditions affect the levels, solubility, and functional state of transcriptional factor Spx and consequently its downstream targets, such as TrfA. We hypothesized the different environmental stimuli may regulated MazEF TAS through YjbH aggregation, soluble Spx, and TrfA (Figure 1).

## Materials and Methods

### Bacteria Cultures, Strains, and Plasmids

All bacteria strains and plasmids used in this work are listed in Table 1. Most *S. aureus* genetic constructs were created in HG003 strain background (Herbert et al., 2010; Sassi et al., 2014). *S. aureus* bacterial cultures were grown on Mueller Hinton Broth (MHB) media until OD_600_ of 0.5–0.7 at 37°C with shaking. Bacteria containing plasmids with chloramphenicol or tetracycline resistance were grown in the presence of 15 μg/ml of chloramphenicol and 3 μg/ml of tetracycline, respectively.

**TABLE 1:** Bacteria strains and plasmids.

*S. aureus* strains					
**Strain**	**Background**	**Strain resistance**	**Plasmid**	**Plasmid resistance**	**Source**

HG003 wt	HG003				(Herbert et al., 2010)
HG003 + pOP173	HG003		pOP173	Cm, Amp	This work
HG003, yjbH:ery 5	HG003	Ery			This work
HG003, yjbH:ery 5 + pOP173	HG003	Ery	pOP173	Cm, Amp	This work
NEWMAN + pCL25-sfGFP	NEWMAN		pCL25-sfGFP	Tet	Gift from Claes von Wachenfeld
NEWMAN + pCL25-sfGFP SAYjbH	NEWMAN		pCL25-sfGFP SAYjbH	Tet	Gift from Claes von Wachenfeld
HG003, Δ*trfA*:tet	HG003	Tet			This work
HG003, Δ*mazEF*	HG003				(Schuster et al., 2015)
HG003, Δ*mazEF* + pAR1884	HG003		pRAB11-MazF	Cm, Amp	This study
HG003, Δ*mazEF* + pOP174	HG003		pOP174	Cm, Amp	This study

**Plasmids**					

**Plasmid name**	**Gene**	**Vector**	***S. aureus* marker**	***E. coli* marker**	**Source**

pOP172 (pUC57-HA3-YjbH)	HA3-YjbH	pUC57		Amp	This work
pOP173 (pMK4-pHU-HA3-YjbH)	HA3-YjbH	pMK4	Cm	Amp	This work
pOP174 (pRAB11)		pRAB11	Cm	Amp	(Helle et al., 2011)
pAR1884 (pRAB11-MazF)	MazF	pRAB11	Cm	Amp	This study

Plasmid pOP172 was obtained from GENEWIZ. Synthetic codon optimized *S. aureus* YjbH gene with HA_3_ tag (HA_3_-YjbH) was synthesized and cloned into pUC57 vector resulting in pOP172. To create pOP173 plasmid, HA_3_-YjbH fragment was *Kpn*I/*Pst*I digested from pOP172 and cloned into pMK4-pHU vector (Andrey et al., 2010). *S. aureus* strains carrying sfGFP alone or sfGFP-YjbH under control of IPTG inducible promoter were kindly provided by Claes von Wachenfeld (Lund University). The pRAB11-MazF plasmid (pAR1884), expressing *mazF* gene under control of anhydrotetracycline (ATc) inducible promoter was constructed by amplification of HG003 *mazF* gene using primers carrying Bgl2 and EcoR1 restriction sites. Amplified product was cut with Bgl2 and EcoR1 and ligated the into pRAB11 vector (Helle et al., 2011). To induce the expression of MazF, 0.2 μM of ATc was added to the cell cultures and incubated for 10–180 min at 37°C with shaking.

### Protein Extraction

Cultures of 25 ml with OD_600_ of 0.7 were collected and washed three times with 1 ml of Phosphate-buffered saline (PBS) buffer. Cells were lysed in the presence of 400 μl of lysis buffer 1 (LB 1) [PBS, 200 μg/ml lysostaphin, 200 μg/ml DNAse I, protease inhibitors (Roche)] for 20 min at 37°C, chilled on ice, and sonicated 10 times with 30-s cycles using Cell Disrupter B-30 (Branson). Extracts were clarified by centrifugation for 10 min at 14000 × *g* 4°C. Total protein concentration was measured in supernatants (SN) by the Bradford protein assay. This method permitted to obtain of about 1 mg of total protein with a concentration of about 2.5–3.0 mg/ml. Samples were mixed with Laemmli Sample Buffer (SB) and analyzed by SDS polyacrylamide gel electrophoresis (SDS-PAGE) with following Coomassie Blue staining or western blot.

### Isolation of Aggregated Proteins

The protocol for isolation of aggregated proteins from *S. aureus* was developed on the base of method described for yeast (Panasenko and Collart, 2012). Cells were grown on MHB until OD_600_ of 0.5–0.8. Control bacterial culture was left at 37°C without treatment. Other cultures were incubated at 37°C for 30 min with 5 mM of diamide, 10% ethanol or with antibiotics [oxacillin (10–40 μg/ml), vancomycin (10–40 μg/ml), kanamycin (50–400 μg/ml), tetracycline (60 μg/ml), erythromycin (20 μg/ml)]. For heat shock (HS), cells were incubated at 53°C for 30 min. After cultures treatment, 15 OD_600_ units were harvested at 4000 g for 5 min, and washed with 1 ml of PBS. Pellets were resuspended in 0.3 ml (20 μl per 1 OD unit) of lysis buffer 2 (LB2) [20 mM Na-phosphate, pH 7.6, 10 mM DTT, 1 mM EDTA, 0.1% Tween 20, 1 mM PMSF, protease inhibitor cocktail (Roche), 200 μg/ml DNAse, 200 μg/ml lysostaphin] and incubated at 37°C for 20 min. Chilled samples were sonicated 10 times with 40-s cycles, using Cell Disrupter B-30 (Branson), and centrifuged for 20 min at 200 × *g* at 4°C. Total protein concentration was measured in supernatants by the Bradford protein assay. Whole cell extracts were adjusted to identical protein concentration of 1.0 mg/ml. 30 μl of supernatants were boiled with 10 μl of 4× SB (total proteins). Anti-Pbp2 antibody was used as a control for the equal protein amount in whole cell extracts. Equal amount of whole cell extracts (200 μl) were centrifuged at 16000 × *g* for 20 min at 4°C to pellet the aggregated proteins. After removing supernatants (soluble fractions), insoluble proteins were washed twice with washing buffer (20 mM Na-phosphate, pH 7.6, 2% of NP-40, 1 mM PMSF, protease inhibitor cocktail (Roche), sonicated (10 s at duty cycle 40%), and centrifuged at 16000 × *g* for 20 min at 4°C. Insoluble (aggregated) proteins were boiled in 50 μl of SB. 60 μl of soluble fractions were boiled with 20 μl of 4× SB. 15 μl of samples were separated by gradient (4–12%) SDS-PAGE, and analyzed by Coomassie Blue staining or western blot.

### Fluorescent Microscopy of sfGFP-YjbH Aggregates

Cells expressing either sfGFP-YjbH or only sfGFP under IPTG inducible promoter were grown on MHB with 3 μg/ml of tetracycline overnight. Cultures were diluted 1/100 in MHB with 3 μg/ml of tetracycline and with 1 mM of IPTG and grown 37°C for 5 h. Cells were incubated at 37°C for 30 min with 5 mM of diamide, or with antibiotics [oxacillin (20–40 μg/ml), tetracycline (20–60 μg/ml), kanamycin (200–400 μg/ml)]. For heat shock (HS) cells were incubated at 53°C for 30 min. Control culture was left at 37°C without treatment. 1.5 ml of cultures were chilled and centrifuged at 4°C 10000 × *g* for 1 min. Pellets were resuspended in 30 μl of PBS and 7 μl of cell suspension was loaded on 1% agar, and analyzed with fluorescent microscope.

### RTqPCR

RNAs were purified using RNeasy Plus Mini Kit (Qiagen) and QIAshredder Kit (Qiagen). DNA was removed using QIAGEN DNase Kit (Qiagen). TaqMan real-time quantitative polymerase chain reaction (RTqPCR) was performed with Platinum Quantitative RT-PCR ThermoScript One-Step System (Invitrogen). All RNAs were tested for the absence of DNA contaminations. Primers and MGB Double-Dye probes for *trfA*, *rsbW*, *16S RNA*, and *gyrB* (Table 2) were designed using Primer Express software (version 1.5; Applied Biosystems), obtained from Eurogentec and used in a concentration 0.05–0.1 μM. For each pair of primers, primer efficiency was calculated and primers and probes were used in concentrations that give the same primer efficiency with housekeeping gene (*16S RNA* or *gyrB*). To quantify RTqPCR data the 2^(−ΔΔCT) method has been used (Rao et al., 2013), where fold change of target gene expression in a target (treated) samples relative to a reference (non-treated) samples was normalized to a reference gene (*16S RNA* or *gyrB*). Thus, the relative gene expression in non-treated samples was set to 1. The errors for the ΔΔCT were obtained by least square error propagation of the standard deviation for the individual RTqPCR measurements performed in triplicates.

**TABLE 2:** RTqPCR primers and probes.

Gene	Type	Name	Sequence
*gyrB*	Forward primer	gyrB-118F	TCAGAGAGAGGTTTGCACCATTT
*gyrB*	Reverse primer	gyrB-185R	CCAGCTAATGCTTCATCGATACTATT
*gyrB*	MGB Double-Dye Probes	gyrB-143P	FAM – TGTGGGAAATTGTCG – MGB Eclipse
*rRNA16S*	Forward primer	rRNA16S-1024F	GATAGAGCCTTCCCCTTCGG
*rRNA16S*	Reverse primer	rRNA16S-1147R	CCGGCAGTCAACTTAGAGTGC
*rRNA16S*	Probe	rRNA16S-1071T	FAM – ACATCTCACGACACGAGCTGACGACA – MGB Eclipse
*rsbW*	Forward primer	rsbW-104F	CACTTTCTGGCGTTTTTTCGA
*rsbW*	Reverse primer	rsbW-167R	GCAATCTTGGCATCTTCAATATCA
*rsbW*	MGB Double-Dye Probes	rsbW-126P	FAM – AGCTGGTGCTACATATG – MGB Eclipse
*trfA*	Forward primer	trfA-314F	AAACATTAGAAGGTGAAGATCAATTAGAAG
*trfA*	Reverse primer	trfA-409R	GTGCTGAAGACTTTTGACGTTT
*trfA*	MGB Double-Dye Probes	trfA-355P	FAM – CAACGAACAAAAGAAAAAGAAGCTCAA – MGB Eclipse

### RNA-Seq Analysis

The induction of the MazF expression by ATc was performed for 10 min. Total RNAs were purified as described above in biological duplicates. Ribosomal RNAs were depleted with RiboZero kit (Illumina). Libraries were created using the Illumina TruSeq stranded mRNA kit. 1st strand cDNAs were synthesized with random primer. Libraries were sequenced at Fasteris SA. Results of RNA-seq were normalized for sequencing depth [normalization estimated by edgeR (Robinson and Oshlack, 2010; Robinson et al., 2010)], and by the length of the gene, in kilobases and presented in RPKM (Reads Per Kilobase Million).

### Antibodies

Anti-HA (anti-influenza hemagglutinin; Sigma) antibodies were used at the dilution 1:3000. Anti-Spx was a gift from Dorte Frees (University of Copenhagen) (Stahlhut et al., 2017) and used in dilution 1:3000. Anti-MazE and anti-MazF antibodies were a gift from Patrick Viollier (University of Geneva) and used in dilution 1:500. Anti-Pbp2 antibodies were a gift from Mariana Gomes de Pinho (ITQB NOVA, University of Lisbon) and used in dilution 1:8000.

## Results

### Environmental Stimuli Lead to Aggregation of the Adaptor Protein YjbH

**FIGURE 2:**
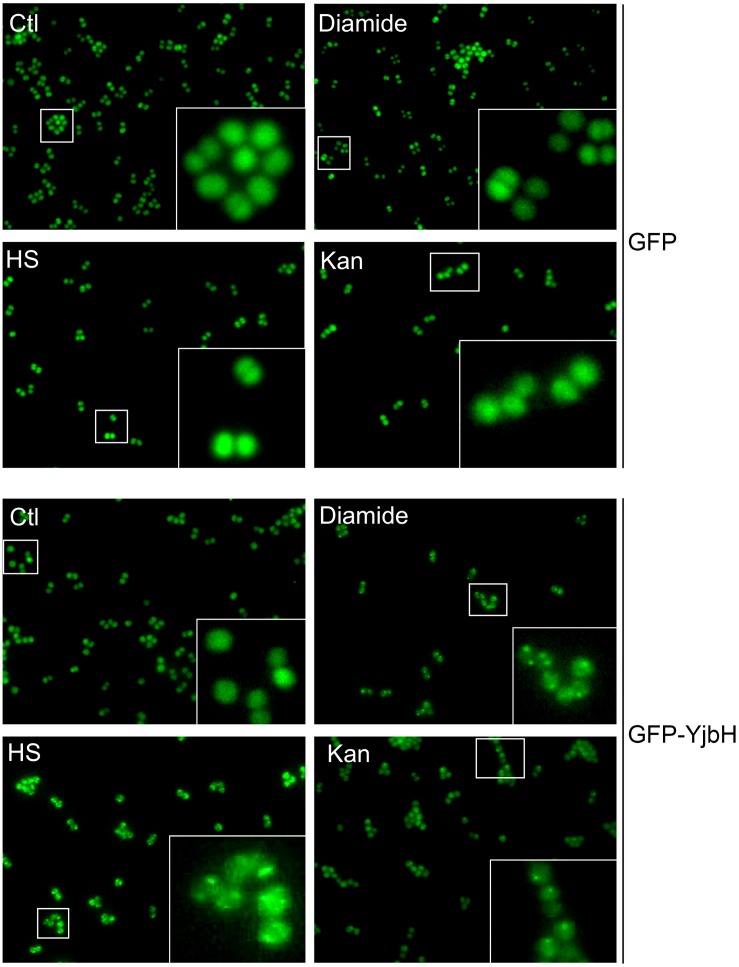
sfGFP-YjbH is accumulated in foci upon heat shock, oxidative stress, and antibiotic treatment. Cells, expressing sfGFP-YjbH were not treated (Ctl), or treated with high temperature (53°C, HS), 5 mM of diamide or 400 μg/ml of ribosome-targeting antibiotic, kanamycin (Kan) and analyzed by fluorescence microscopy (lower panel, GFP-YjbH). Cells, expressing only sfGFP were used as a control and treated in the same manner (upper panel, GFP).

MazE antitoxin is regulated by an upstream pathway activated by yet unknown stimuli and unknown mechanisms (Figure 1). We hypothesized that YjbH adaptor protein may be involved in stimulus sensing. In *B. subtilis* it was demonstrated that YjbH is aggregated upon heat shock and diamide-induced oxidative stress, and this aggregation is accompanied by an increase in level of transcriptional regulator Spx (Larsson et al., 2007; Engman and von Wachenfeldt, 2015). We first asked whether YjbH in *S. aureus* is also prone to aggregation and if so, in which conditions. We used cells expressing IPTG inducible sfGFP alone or sfGFP fused to YjbH (sfGFP-YjbH). After IPTG-induction of sfGFP constructs, bacterial cells were subject to treatment with heat shock, diamide and ribosome-targeting antibiotic, kanamycin, and observed with the fluorescent microscopy (Figure 2). A bacterial strain carrying sfGFP alone show a fluorescent signal distributed homogeneously in all cells before and after tested conditions (Figure 2, upper panel, GFP). In non-treated cells, expressing sfGFP-YjbH fusion, fluorescent signal was also distributed homogeneously. In contrast, distinct fluorescent foci were visible after 30 min of treatment with diamide, heat shock, and kanamycin (Figure 2, lower panel, GFP-YjbH). The same foci were detected after treatment with the cell wall antibiotic, oxacillin, and ribosome-targeting antibiotic, tetracycline (data are not shown).

We speculate that visible foci appeared after stress treatment are aggregated sfGFP-YjbH protein, as was described for *B. subtilis* (Engman and von Wachenfeldt, 2015). To verify this hypothesis, we developed a method to isolate insoluble proteins in *S. aureus*. We treated wild-type cells with different stress conditions, such as heat shock (53°C), ethanol (10%), diamide (5 mM), and different antibiotics. Whole cell extracts were adjusted to the same protein concentration. Equal amounts of total protein were separated into soluble and insoluble fractions by centrifugation. Precipitated proteins were washed twice by sonication in buffer containing 2% of non-ionic detergent NP-40 to disrupt the membranes. Proteins remained insoluble after that treatment, are likely to be aggregated proteins (Engman and von Wachenfeldt, 2015), therefore we use the term aggregates for them. Insoluble proteins were resuspended in the sample buffer containing 2% of SDS and analyzed by SDS-PAGE and Coomassie Blue staining.

**FIGURE 3:**
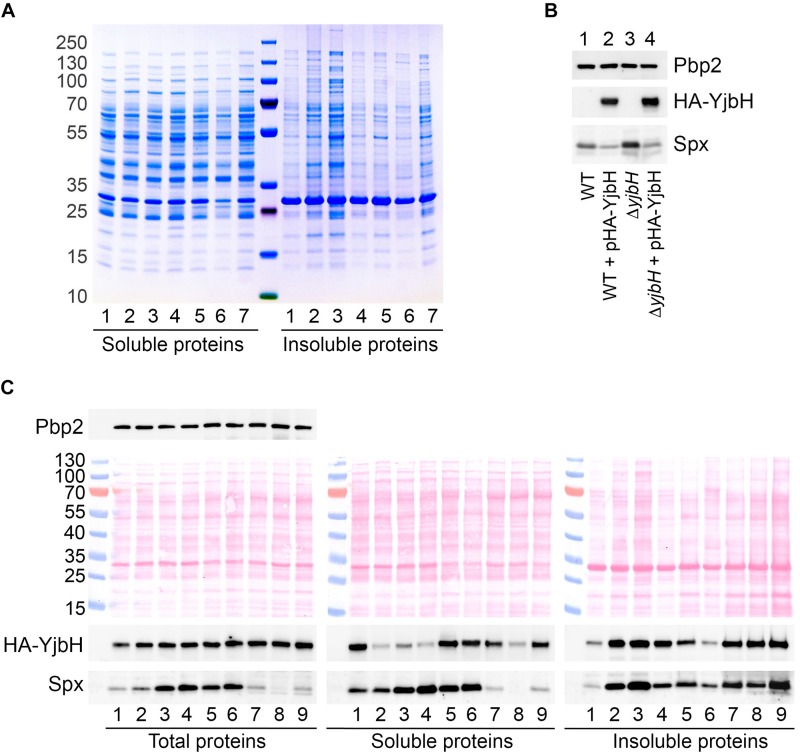
HA-YjbH is aggregated upon heat shock, oxidative stress and antibiotic treatment. **(A)** Exponentially growing cells (1) were treated with high temperature of 53°C (2), 10% of ethanol (3), 5 mM of diamide (4), 10 μg/ml of oxacillin (5), 10 μg/ml of vancomycin (6), and 50 μg/ml of kanamycin (7). Whole cell extracts were adjusted to the same total protein concentration and fractionated into the soluble and insoluble fractions. Samples were separated by gradient (4–12%) SDS-PAGE, and analyzed by Coomassie Blue staining. **(B)** Cells, either wild type (WT) or Δ*yjbH* were transformed with plasmid expressing HA-tagged YjbH (HA-YjbH). Whole cell extracts were prepared and the levels of HA-YjbH and Spx were estimated by western blot with anti-HA and anti-Spx antibodies. Penicillin binding protein 2, Pbp2, was used as a control for equal loading. **(C)** Δ*yjbH* cells expressing HA-YjbH were not treated (1) or treated with high temperature (53°C) (2), 10% of ethanol (3), 5 mM of diamide (4), 40 μg/ml of oxacillin (5), 40 μg/ml of vancomycin (6), 400 μg/ml of kanamycin (7), 60 μg/ml of tetracycline (8), and 20 μg/ml of erythromycin (9). Whole cell extracts were adjusted to the equal protein concentration and fractionated into the soluble and insoluble fractions. Proteins were separated by gradient (4–12%) SDS-PAGE and transferred to the nitrocellulose membranes. Membranes were stained with Ponceau S for detection of protein bands and control of loading, and analyzed by western blot with anti-HA, anti-Spx, and anti-Pbp2 antibodies. Pbp2 was used as a control for the equal protein amount in whole cell extracts. Western blots were quantified with ImageJ program and normalized to the signal in untreated control (Supplementary Figure S1).

No difference was observed in soluble protein fractions from non-stress or stress conditions (Figure 3A). However, significant difference was in insoluble fractions. Heat shock, ethanol and kanamycin, induced massive protein aggregation compared to non-stressed conditions, suggesting that under these conditions many proteins are altered and prone to aggregation. Oxidative stress induced by diamide, and treatment with cell wall antibiotics oxacillin and vancomycin, did not lead to notable protein aggregation.

To prove that visible foci at Figure 2 are indeed insoluble sfGFP-YjbH, we constructed a plasmid expressing HA-tagged YjbH protein expressed from a constitutive HU promoter. The plasmid was transformed into either wild-type cells or cells deleted for *yjbH* (Δ*yjbH*). HA-YjbH expression was detected in both strains (Figure 3B). Spx accumulation was observed upon *yjbH* deletion, as previously described in *B. subtilis* (Larsson et al., 2007) and in *S. aureus* (Renzoni et al., 2011). Spx level was decreased upon YjbH plasmid overexpression (Figure 3B). Thus, HA-tagged YjbH protein was indeed functional and compensated the *yjbH* deletion.

The effect of different environmental stresses on YjbH protein levels was then analyzed. Exponentially growing Δ*yjbH* bacteria expressing HA-YjbH were subjected to the different stresses, such as heat, ethanol, diamide and antibiotics. Total protein extracts were adjusted to the same protein concentration and fractionated. Fractions of soluble and insoluble proteins were analyzed by SDS-PAGE and western blot for the presence of YjbH and Spx.

In total protein extracts the levels of YjbH were not changed under stress to non-stress conditions (Figure 3C and Supplementary Figure S1, Total proteins). However, the distribution of YjbH in soluble and insoluble fraction was affected (Figure 3C and Supplementary Figure S1, Soluble and Insoluble proteins). In non-stressed conditions YjbH mainly remained soluble (Figure 3C, lane 1). Only a minor fraction of YjbH was insoluble. In contrast, a significant amount of YjbH was insoluble after exposure to heat shock, ethanol, diamide, and ribosome-targeting antibiotics, such as kanamycin, tetracycline, and erythromycin (Figure 3C, lanes 2–4, 7–9). At the same time, less YjbH remained in soluble fraction under these conditions. It was shown earlier, that insoluble YjbH is not membrane associated but rather an aggregate (Engman and von Wachenfeldt, 2015). Cell wall antibiotics, oxacillin and vancomycin, did not cause significant YjbH aggregation and it remained mainly soluble (Figure 3C, lanes 5–6). Taken together, these observations suggest that in *S. aureus* YjbH is prone to aggregation upon environmental stimuli like it does in *B. subtilis*. However, not all tested stress conditions induced similar YjbH aggregation. Importantly, YjbH aggregation was observe under oxidative stress caused by diamide that did not result in dramatic increase of general protein aggregation and looked similar to untreated conditions (Figure 3A, right, lanes 1 and 4). Thus, YjbH aggregation may be selective and exploited to regulate certain environmental responses.

### Transcription of *trfA* Depends Not Only on YjbH Solubility but Also on Functional State of Spx

In *B. subtilis* heat and diamide induced YjbH aggregation and consequent Spx stabilization was observed due to decreased proteolysis of Spx by ClpXP (Garg et al., 2009; Engman and von Wachenfeldt, 2015). To analyze if stress conditions resulting in YjbH aggregation in *S. aureus* leads to accumulation of Spx protein, we followed steady state Spx protein levels by western blot. We observed increased levels of Spx in case of ethanol and diamide treatment (Figure 3C, Total proteins, lanes 3 and 4), when YjbH was aggregated (Figure 3C, Insoluble proteins, lanes 3 and 4). However, upon oxacillin and vancomycin treatment we also observed increased levels of Spx (Figure 3C, Total proteins, lanes 5 and 6) while YjbH remains mainly soluble in these conditions (Figure 3C, Soluble proteins, lanes 5 and 6). At the same time, heat shock and ribosome-targeting antibiotics, that caused aggregation of YjbH, did not lead to increased levels of Spx in total extracts (Figure 3C, Total proteins, lanes 2, 7–9). Thus, not all stress conditions leading to YjbH aggregation, resulted in Spx increased levels. This observation suggests that Spx steady state levels are not exclusively modulated by YjbH.

To understand better the link between YjbH solubility and Spx levels we next analyzed the distribution of Spx between soluble and insoluble fractions during various stresses. We found that even if the total amount of Spx was not induced upon heat shock or ribosome-targeting antibiotics, the majority of protein was found in aggregates and a very low amount was detected in soluble fractions [Figure 3C, compare Soluble and Insoluble fractions (lanes 2, 7–9)]. Under diamide, oxacillin or vancomycin stresses, where increased Spx levels were observed in the total extracts, also higher amounts were found in the soluble fraction compared to the aggregated fraction [Figure 3C, compare Soluble and Insoluble fractions (lanes 4–6)]. These results demonstrate that diamide and cell wall antibiotics, oxacillin and vancomycin, increase the total levels of Spx. Spx mostly remains soluble and, thus, probably functional under these stress conditions. Heat shock and ribosome-targeting antibiotics, kanamycin, tetracycline, and erythromycin, treatments did not increase total levels of Spx, and Spx was found aggregated in probably an inactive form.

**FIGURE 4:**
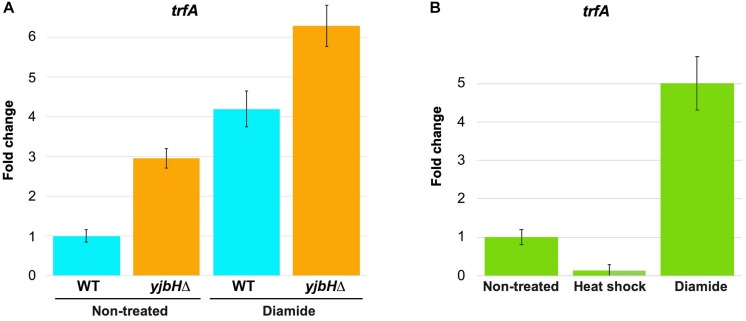
Transcription of *trfA* correspond to the high protein levels and solubility of Spx. RNAs were purified from cells described in Figures 3B,C. The level of *trfA* RNA was measured by RTqPCR in non-treated cells and in cells treated for 30 min with either heat shock or oxidative stress induced by diamide. *16S RNA* was used as a reference normalizing housekeeping gene. Fold change of *trfA* gene expression in treated samples relative to non-treated samples was normalized to a reference gene (*16S RNA*). The relative gene expression in non-treated cells was set to 1. **(A)** The levels of *trfA* were increased in the *yjbH* deletion mutant, where the levels of Spx were increased. **(B)** Transcription of *trfA* is decreased after heat shock and increased after oxidative stress, that correspond to solubility of Spx.

Spx is a transcriptional factor, which interacts with the alpha−subunit of the RNA polymerase and induces transcription of many genes, including *trfA* (Jousselin et al., 2013). In *B. subtilis* it was shown that oxidation of Spx molecule leads to formation of intramolecular S-S bond between Cys10 and Cys13, that modulates the activity of Spx (Antelmann and Helmann, 2011; Rojas-Tapias and Helmann, 2018). These amino acid residues are conserved in *S. aureus*. However, it is unknown how Spx oxidation or aggregation may affect its activity in *S. aureus*. To determine how functional was Spx upon stress conditions we followed its activity by analyzing *trfA* transcription using RTqPCR. We analyzed expression of *trfA* transcript in conditions that cause increased Spx protein levels, such as *yjbH* deletion, and probably modulate Spx activity, such as diamide. RNAs were purified from diamide treated and non-treated cells, and *trfA* mRNA levels were measured by RTqPCR. In non-treated conditions we observed an increase of *trfA* transcript levels in the *yjbH* deletion mutant compared to the wild-type cells (about three times) (Figure 4A). Diamide treatment increases *trfA* transcription in both wild-type and *yjbH* deleted strains. However, the difference in *trfA* transcription in wild type and *yjbH* deletion mutant on diamide was less, that in untreated conditions (4.2 and 6.2 compare to 3 and 1). Diamide treatment led to higher induction of *trfA* in wild-type cells compare to Δ*yjbH* cells (Figure 4A, blue columns 1 and 4.2 compare to orange columns 3 and 6.2). These data are expected, because deletion of *yjbH* results only in increase of Spx protein levels, while, diamide treatment results in both, increase of Spx protein levels and oxidation of Spx protein, leading to higher levels of active Spx and consequently, higher *trfA* transcription.

Next, we tested if solubility affects functional state of Spx. We analyzed expression of *trfA* under heat and oxidative stress, where we observed different solubility of Spx. We found, that *trfA* mRNA levels were decreased upon heat shock stress while increased upon oxidative stress (Figure 4B). These observations corroborate our hypothesis that Spx is aggregated and less functional after heat shock, but not after diamide treatment where Spx was found soluble.

### Oxidative Stress Decreases MazE Levels and Increases MazF Activity That Correlates With Solubility of YjbH and Spx

The correlations between YjbH aggregation, protein levels of active/inactive Spx and *trfA* transcription after heat or oxidative stresses, predict a different end-point effect on MazE antitoxin levels and MazF toxin activity. We, therefore, analyzed YjbH, Spx and MazE protein levels after diamide or heat shock treatment at different time points. Soluble and insoluble proteins were isolated from cell extracts adjusted to the same total protein concentrations.

**FIGURE 5:**
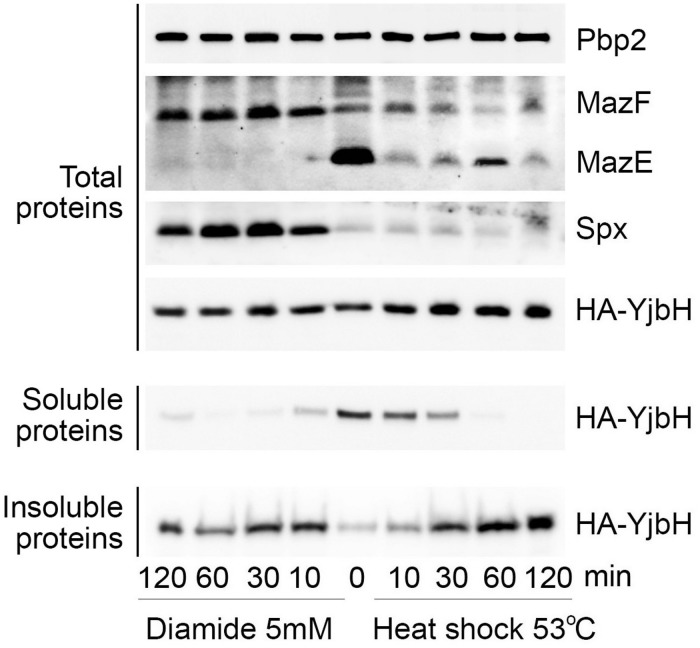
Upon heat shock and oxidative stresses MazE levels correlate with YjbH aggregation and Spx solubility. Δ*yjbH* cells expressing HA-YjbH were treated with high temperature (53°C, right), or with 5 mM of diamide (left) for different times, as indicated. Non-treated cells are marked by time 0. Whole cell extracts (Total protein) were adjusted to the same protein concentration and soluble and insoluble proteins were isolated. Samples were separated by gradient (4–12%) SDS-PAGE and analyzed by western blot with anti-HA, anti-Spx, anti-MazE, and anti-MazF antibodies. Pbp2 was used as a control for the equal protein amount in whole cell extracts.

In total protein extracts the levels of YjbH were similar before and after both stress conditions (Figure 5, Total proteins). YjbH was mostly found in the soluble fractions before stress (time 0). After 10 min of diamide treatment YjbH aggregated and, concomitantly, Spx was stabilized (Figure 5, left panel, diamide, Spx in Total proteins). As diamide treatment induces *trfA* transcription through increased amounts of functional Spx (Figure 4B), we expected an increase of MazE proteolysis. Indeed, after 10 min of diamide treatment we observed a dramatic reduction of MazE protein levels suggesting an increase of *trfA*-dependent MazE proteolysis by ClpCP (Figure 5, left panel, diamide, MazE in Total proteins). At the same time upon diamide treatment the level of MazF was increased, compared to untreated cells (Figure 5, left panel, diamide, MazF in Total proteins).

After heat shock YjbH remained soluble for longer than upon diamide stress, but finally aggregated after 60 min of treatment (Figure 5, right panel, YjbH in Soluble and Insoluble proteins). Despite YjbH aggregation, Spx levels remained low and unchanged during all times of heat shock treatment compared to non-stressed conditions (Figure 5, right panel, heat shock, Spx in Total proteins). In agreement with inactive Spx that was found mostly aggregated after heat shock (Figure 3C), we observed a consequent reduced transcription of *trfA* gene (Figure 4B). A decreased *trfA* transcription suggests stabilization of MazE antitoxin, as reduction of TrfA will reduce MazE proteolysis. However, we did not observe stabilization of MazE proteolysis after heat shock. Levels of MazE protein were reduced compared to non-stress conditions, but not as much as after diamide treatment (Figure 5, right panel, MazE in Total proteins). These observations suggest that other potential factors must therefore be affected by heat shock and that could explain reduced MazE protein levels without an increase of TrfA.

**FIGURE 6:**
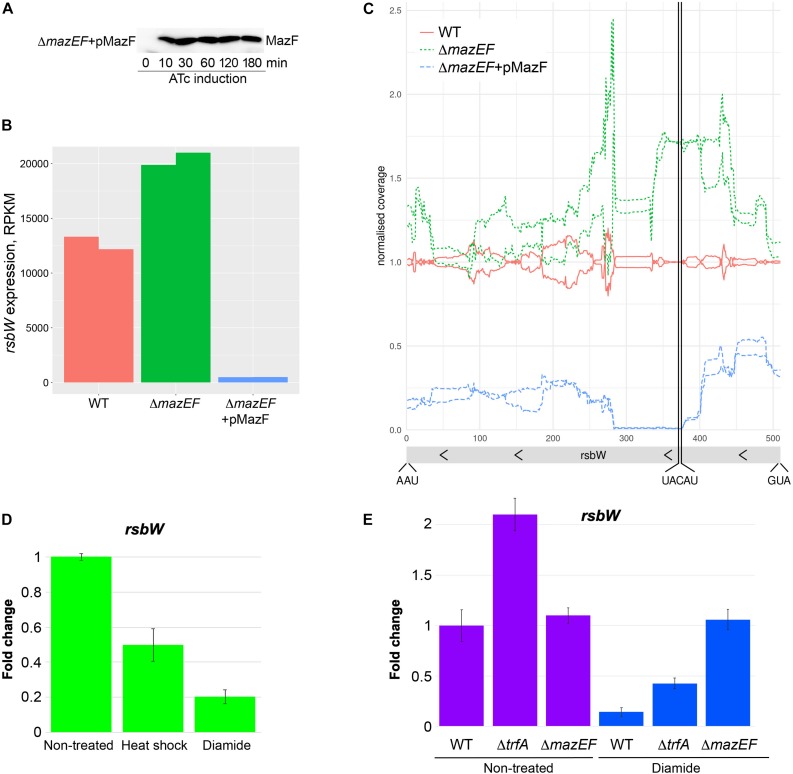
MazF cleaves *rsbW* at UACAU cleavage site and MazF activity correlates with YjbH aggregation and Spx solubility. **(A)** Western blot analysis of MazF protein produced after anhydrotetracycline (ATc) induction. The *mazEF* deleted strain was transformed with the pRAB11-MazF plasmid (indicated as pMazF), expressing *mazF* gene under control of ATc inducible promoter. Cultures were collected before ATc induction (time 0). Then ATc was added and cultures were collected after 10, 30, 60, 120, and 180 min. Protein were extracted and analyzed by SDS-PAGE and western blot with anti-MazF antibody. **(B)** Effect of MazF on *rsbW* level. Wild-type or Δ*mazEF* cells expressing either empty vector (WT and Δ*mazEF*) or *mazF* gene under control of ATc promoter (Δ*mazEF* + pMazF) were collected after 10 min of ATc induction. Total RNAs were purified and analyzed by RNA-seq. The RPKM values (Reads Per Kilobase Million) normalized by edgeR after excluding plasmid genes are show in duplicates. **(C)** In-depth analysis of *rsbW* gene expression by RNA-seq. Cells were treated as in panel **(B)**. RNA-seq coverage along the *rsbW* region relative to the average wild type is shown. The raw RNASeq coverage was normalized to the total number of reads over the chromosome region and then divided by the coverage of the wild-type samples. The start codon, stop codon and, MazF cleavage site are indicated. **(D)** RNAs were purified from cells described in Figure 5. The level of *rsbW* RNA was measured by RTqPCR in non-treated cells and in cells treated for 30 min with either heat shock or oxidative stress induced by diamide. *gyrB* was used as a reference normalizing housekeeping gene. Fold change of *rsbW* gene expression in treated samples relative to non-treated samples was normalized to a reference gene (*gyrB*). The relative gene expression in non-treated cells was set to 1. **(E)** The level of *rsbW* RNA was measured by RTqPCR in wild-type, Δ*trfA* or Δ*mazEF* cells treated or not with diamide for 30 min, as described in panel **(D)**. The relative gene expression in non-treated wild-type cells was set to 1.

The decreased MazE levels upon diamide or heat shock stress predict not only stabilized MazF, but also a higher MazF endoribonuclease activity compared to non-stressed condition. To investigate MazF activity we created a plasmid expressing MazF under control of inducible promoter. We transformed this plasmid in the strain deleted for *mazEF* TAS to model the situation when MazF is not inhibited by MazE. MazF protein was visible after 10 min of induction (Figure 6A). To investigate MazF endoribonuclease activity we analyzed mRNA levels of *rsbW* gene, one of the previously described MazF endoribonuclease targets (Schuster et al., 2015). We expect a higher cleavage or decreased mRNA levels of *rsbW* upon MazF toxin expression or conditions increasing MazF activity. The level of *rsbW* transcript was slightly increased in Δ*mazEF* cells compared to wild-type cells, and it was dramatically decreased when *mazF* transcription was induced in Δ*mazEF* cells (Figure 6B). In-depth analysis of reads covering *rsbW* transcript, clearly showed a reduction of reads mapping the identified MazF cleavage motif (UACAU) present in *rsbW* gene (Figure 6C). These results corroborate previous observations that *rsbW* mRNA is a target of MazF toxin (Schuster et al., 2015) and show that *rsbW* transcript levels are decreased upon MazF overexpression.

To analyze if diamide or heat shock stress affect MazF endoribonuclease activity, we measure levels of *rsbW* mRNA target by RTqPCR using a probe hybridizing exactly on the MazF cleavage motif. For control and normalization, we used *gyrB* gene that was previously shown to be a non-target of MazF (Fu et al., 2009). We observed two-fold and a five-fold reduction of *rsbW* mRNA levels upon heat shock or diamide treatment, respectively (Figure 6D).

Taken together, these results show that decreased levels of MazE antitoxin upon diamide and heat shock resulted in modulation of MazF toxin activity. The slight decrease of *rsbW* mRNA cleavage, despite the reduced levels of MazE upon heat shock, can be explained by residual MazE sufficient to maintain MazF inactive. In contrast, MazE levels upon diamide are absent or highly reduced thus resulting in liberated highly active MazF toxin.

To show that decreased levels of MazE protein upon diamide treatment (Figure 5), affecting MazF activity, is dependent on *trfA* adaptor, we analyzed *rsbW* levels in *trfA* deleted strain with or without diamide treatment (Figure 6E). As expected, the deletion of *trfA* resulted in increased levels of *rsbW* in both conditions, confirming the role of TrfA in MazE degradation and, consequently, MazF activation. We further analyzed whether the deletion of *mazEF* affects *rsbW* levels in cells treated or not with diamide. Deletion of *mazEF* in non-treated conditions did not dramatically change *rsbW* levels, being in agreement with data obtained by RNA-seq (Figure 6B). These results were expected as MazF activity is inhibited by MazE in wild type or not present in Δ*mazEF* mutant. However, upon diamide treatment we observed strong stabilization of *rsbW* in Δ*mazEF* mutant, that may be explained by some additional effects of diamide on *rsbW* expression by unknown mechanisms.

## Discussion

MazE antitoxin level ensures formation of at least a stoichiometric toxin-antitoxin complex and consequently, MazF inactivity. Under unknown conditions, the MazF endoribonuclease activity can increase and result in destruction of mRNA molecules leading to growth stasis (reviewed in Sierra et al., 2019). The clinical importance of MazF was recently highlighted by studies showing the role of MazF expression in chronic *S. aureus* infections (Ma et al., 2019). Using a murine abscess model of infection, MazF expression was shown to inhibit biofilm formation and to increase antibiotic tolerance allowing transition of *S. aureus* from acute to chronic infections (Ma et al., 2019). However, it is still unknown how *S. aureus* induces MazF expression and activity under *in vivo* infection conditions. To understand the possible mechanisms of MazF-activation, we investigated how MazF is regulated under different growth conditions.

In *S. aureus*, evidence suggests that the dormancy-related MazEF TAS is controlled by two different upstream proteolytic systems (ClpCP and ClpXP) involving two adaptor proteins, TrfA and YjbH, respectively. As YjbH adaptor was shown in *B. subtilis* to be prone to aggregation (Engman and von Wachenfeldt, 2015), we postulated that modulation of YjbH activity through aggregation can be a key step to control MazEF-dependent hdormancy (Figure 1). Indeed, we showed that upon various environmental stress stimulus *S. aureus* YjbH also aggregates and induces downstream effects regulating MazEF TAS through Spx/TrfA pathway. We showed that diamide induced oxidative stress, causes an increase of MazF toxin activity. Specifically, we uncovered a diamide-stress modulation of MazF activity through an upstream pathway that involves YjbH aggregation, accumulation of active/soluble Spx and increase transcription of the ClpCP adaptor protein TrfA. Increase of *trfA* transcription upon antibiotic treatment is accompanied by an increase of TrfA protein levels (Jousselin et al., 2013). And the decreased protein levels of TrfA are correlated with the increased protein levels of MazE (Donegan et al., 2014). In agreement with these previous studies, we showed, that the TrfA increase leads to decreased levels of MazE antitoxin and higher amounts of active MazF toxin. These results show that at least oxidative stress increases MazF activity, a stress condition certainly found during *S. aureus* eukaryotic cell infection.

Despite a decrease in MazE antitoxin levels upon heat shock there was only a slight decrease in MazF activity. Since, the level of MazF was also decreased upon heat shock, we suggested that the low amount of MazE antitoxin present was sufficient to maintain low level of MazF inactive. Interestingly, heat shock reduction of MazE levels was achieved by upstream changes that do not affect Spx accumulation in contrast to diamide stress. Heat shock did not induce accumulation of Spx protein but instead resulted in higher amounts of aggregated and inactive Spx, in accordance with decreased transcription of *trfA*. A potential decreased activity of ClpCP proteolytic systems at heat shock may explain TrfA stabilization that assists MazE degradation.

We observed that not all conditions leading to YjbH aggregation resulted in Spx accumulation. This finding can be explained by stimuli-specific changes in other proteolytic partners involved in Spx degradation. Indeed, Spx accumulation upon diamide stress in *B. subtilis* is dependent on decreased activity of ClpP protease and aggregation of both ClpX and YjbH proteins (Garg et al., 2009; Engman and von Wachenfeldt, 2015). Moreover, it has recently been observed that depending on the stimuli, different regulatory events occur to increase and activate Spx (Rojas-Tapias and Helmann, 2018). While activation of *Spx* regulon under oxidative stress is accompanied by decreased proteolysis and decreased oxidation state of Spx itself, activation of *Spx* regulon under cell wall stress requires *Spx* transcriptional increase and Spx reduced state (Rojas-Tapias and Helmann, 2018).

Our observations suggest that a similar stimuli-dependent effect is present in *S. aureus*. We observed an increased accumulation of Spx protein both under diamide and vancomycin stress, however, achieved through different regulatory events. Diamide induces YjbH aggregation and no effect on *Spx* transcription was observed (our unpublished observation). In contrast, vancomycin does not induce YjbH aggregation but induces *Spx* transcription (our unpublished observation). Interestingly, both conditions result in production of an active Spx because transcription of Spx targets, such as *trfA*, is increased (Jousselin et al., 2013).

We argue that adaptor proteins YjbH and TrfA, proteolytic systems ClpXP and ClpCP and, finally, Spx transcriptional regulator are involved in upstream control of MazEF TAS. However, expression and activity of YjbH and TrfA, ClpXP and ClpCP, and Spx can be differently modulated, as it was observed previously upon diamide and heat shock in *B. subtilis* and *S. aureus* (Frees et al., 2003, 2004; Zhang and Zuber, 2007; Garg et al., 2009). YjbH can be controlled at both transcriptional and/or post-transcriptional levels. Oxidative stress induced by diamide, activated transcription of *yjbH* in *S. aureus* (Engman et al., 2012). However, no other stress stimuli have been tested. At the post-transcriptional level, YjbH activity can be modulated by aggregation and/or through anti-adaptor proteins and/or phosphorylation following different stimuli, as shown in *B. subtilis* and other bacteria (Kirstein et al., 2007; Elsholz et al., 2011; Engman and von Wachenfeldt, 2015; Rojas-Tapias and Helmann, 2018). Our results show that in *S. aureus*, YjbH can also aggregate, however, the identification of YjbH anti-adaptors such as YirB in *B. subtilis*, or adaptor phosphorylation remains to be investigated. Also, no studies have been conducted to investigate potential anti-adaptors or phosphorylation of TrfA, the second adaptor protein governing MazEF TAS.

Finally, some studies have analyzed the effect of different stress stimuli on Spx. First, *Spx* is transcribed from at least two different promoters and affected by mutation of *clpP* and *clpX* genes (Pamp et al., 2006; Jousselin et al., 2013). Conditions inducing Spx protein accumulation reduced *Spx* transcription, thus, *Spx* was proposed to be a repressor of its own expression (Pamp et al., 2006; Donegan et al., 2019). Interestingly, *Spx* transcription is induced by anaerobiosis and not affected by high temperature, diamide or NaCl (Pamp et al., 2006). In agreement with these observations, we did not observe increased *Spx* transcription under diamide, heat shock, oxacillin or kanamycin stress while a two-fold induction was observed upon vancomycin stress [(Jousselin et al., 2013) (and our unpublished observation)]. Secondly, Spx is not regulated only at the transcriptional level. As no correlation between transcription and Spx protein levels was observed, Spx post-transcriptional regulation was postulated. Indeed, Spx levels can be modulated by proteolysis through ClpXP, and Spx activity can be modulated by its redox state that differs upon different stimuli in *B. subtilis* (Rojas-Tapias and Helmann, 2018). We observed a stimuli dependent Spx protein aggregation that affected Spx activity, and could conceivably be responsible for downstream effects, such as regulation of MazEF TAS.

## Conclusion

In conclusion, the exact mechanisms of sensing and signal transmission that culminate in MazEF TAS activation remain to be elucidated. Our work demonstrates YjbH aggregation in *S. aureus* in response to different environmental stresses, may presumably lead to modulation of MazF activity. We found oxidative stress as one potential *in vivo* growth condition regulating MazF activity. Our observation supports the hypothesis that oxidative stress, found upon *in vivo* infection in *Escherichia coli*, can trigger activation of MazF endonuclease leading to a reduced metabolic state and, consequently, entry into persistence state (Mok et al., 2015). Our hypothesis was strongly supported by the very interesting recent study where the persister-like phenotype in *B. subtilis* was connected to increased levels of Spx at specific environmental conditions, such as stationary phase (Schafer et al., 2019).

## Data Availability Statement

The datasets generated for this study are available on request to the corresponding author.

## Author Contributions

OP designed and performed the experiments, constructed the plasmids and strains, and participated in the writing of the manuscript and conception of the work. OK did the fluorescent microscopy. AR participated in the writing of the manuscript and conception of the work. FB did all the RNAseq analysis and the analysis of MazF cleavage site, and participated in the discussion of the results, revision of the manuscript, writing of the revised manuscript, and conception of the work.

## Conflict of Interest

The authors declare that the research was conducted in the absence of any commercial or financial relationships that could be construed as a potential conflict of interest.
