# Supplementary material for: YjbH Solubility Controls Spx in Staphylococcus aureus: Implication for MazEF Toxin-Antitoxin System Regulation
Source: Front Microbiol. 2020 Feb 6;11:113. doi: 10.3389/fmicb.2020.00113 (PMC7016130; doi:10.3389/fmicb.2020.00113)
Supplement: Supplementary file 1 [file Data_Sheet_1.PDF]

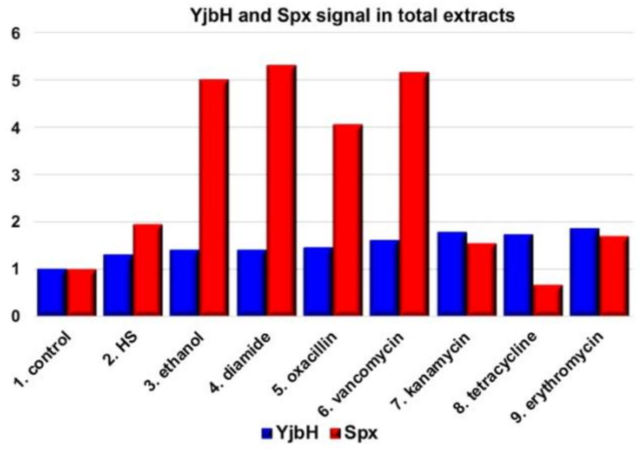

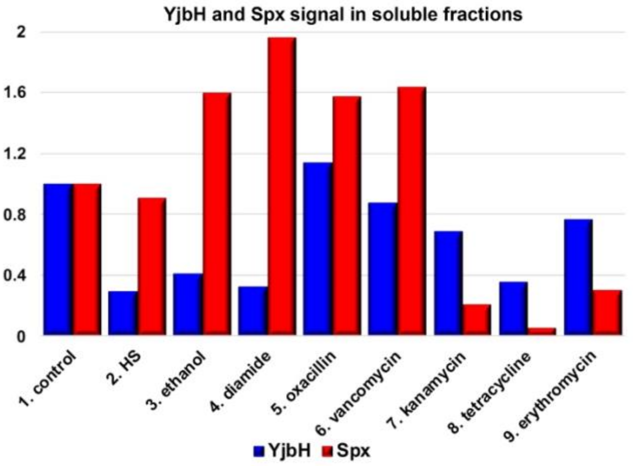

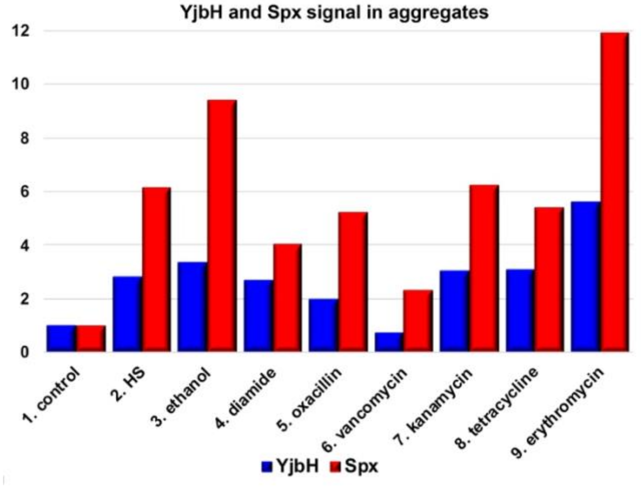

Figure S1. Quantification of the western blots presented on figure 3C. Western blots present on Figure 3C were quantified with ImageJ program and normalized to the signal in untreated control to show the relative amounts.
